# Benign Chondroid Syringoma on Chin: A Case Report and Literature Review

**DOI:** 10.7759/cureus.34571

**Published:** 2023-02-02

**Authors:** Garima Anandani, Yashdeep Singh Pathania, Tarang Patel, Riddhi Parmar, Gauravi Dhruva, CDS Katoch

**Affiliations:** 1 Pathology Department, All India Institute of Medical Sciences, Rajkot (AIIMS Rajkot), Rajkot, IND; 2 Dermatology Department, All India Institute of Medical Sciences, Rajkot (AIIMS Rajkot), Rajkot, IND; 3 Pathology, Pandit Deendayal Upadhyay (PDU) Medical College, Rajkot, IND; 4 Pulmonology Department, All India Institute of Medical Sciences, Rajkot (AIIMS Rajkot), Rajkot, IND

**Keywords:** multiple components, epithelial and mesenchymal, mixed tumour, benign skin adnexal tumour, chondroid syringoma

## Abstract

Chondroid syringoma is a cutaneous adnexal tumor originating from sweat glands origin. It is rare in occurrence and usually benign, having an incidence of 0.01 to 0.098%. As these tumors are uncommon, their diagnosis is missed many times and are misdiagnosed. Hence in any case of facial skin swelling increasing slowly in size, this entity should be kept in mind as one of the possibilities and differential diagnosis. Histopathological examination of the excision biopsy gives the definitive confirmatory diagnosis. Surgically excising the swelling locally along with a surrounding normal tissue cuff is the standard treatment given which prevents recurrence. Hereby we present a 35-year-old case of facial chondroid syringoma having a focal component of eccrine hidrocystoma, keratinous cyst as well as syringocystadenoma papilliferum on the chin that was clinically suspected to be an epidermoid cyst or mucocele.

## Introduction

Chondroid syringoma (CS) is a skin adnexal tumor with a very low incidence ranging from 0.01% to 0.098% of all the primary skin neoplasms. It is a sweat gland tumor, usually benign, alias mixed tumor of the skin, comprised of epithelial as well as mesenchymal elements [1]. Head and neck is the most frequent site of occurrence for these unusual tumors [2]. They usually occur between 20 to 60 years of age with a male predisposition [3]. 

Clinically it presents as a firm, nodular lesion which is painless, increasing slowly in size, attached to the overlying skin but not the deeper tissue [4]. CS is asymptomatic per se but the patients present with localised symptoms based on its site of occurrence. For example, in a case of CS of intraocular region, there was exophthalmos leading to ocular changes and symptoms due to increased pressure [5]. Most of these cases are benign, however, they are predisposed to malignant transformation, as well as metastasis and recurrence. The extremities are the usual sites for these malignant cases unlike the usual slow-growing nodule in head and neck region of their benign counterpart [6,7].

## Case presentation

A 35-year-old male presented with a history of swelling over the chin below the lower lip measuring 1.5x1.0 cm. It was non-tender and soft to firm in consistency. The overlying skin was greyish-brown in colour and adherent to the swelling. Intraoral examination showed no vestibular edema and no obvious intraoral pathology. The swelling had been growing steadily for the past two years, but remained asymptomatic otherwise. The patient sought treatment only for cosmetic reasons and had consulted at various hospitals previously where it was labelled as folliculitis and treated accordingly. However there was no improvement, so he came for consultation in the Outpatient Department (OPD) of Dermatology in our hospital. Herein the probable clinical diagnoses were either epidermoid cyst or mucocele. 

**Figure 1:**
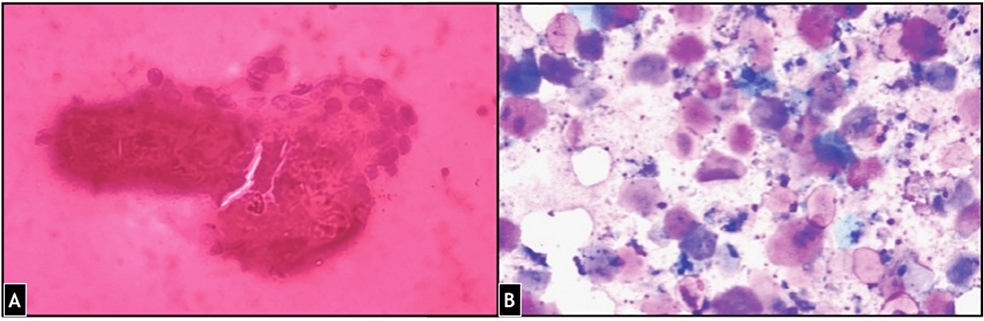
FNAC smears from the lesion show scattered nucleate and anucleate squamous cells and adnexa in keratinous background. (A) Geimsa stain 400X (B) PAP stain 400X FNAC: Fine needle aspiration cytology, PAP: Papanicolaou

Fine needle aspiration cytology (FNAC) was performed. Blood mixed with sticky fluid was aspirated. Smears showed abundant macrophages, scattered mature squamous cells, anucleated squames, few neutrophils and lymphocytes in hemorrhagic and keratinous background (Figure 1). This was suggestive of an epidermoid cyst and histopathological examination was advised for further evaluation.

**Figure 2:**
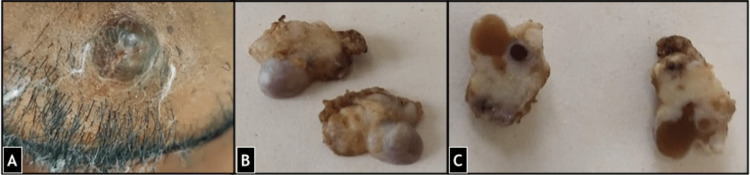
(A) The lesion was present on the chin with greyish brown overlying skin. (B) Nodular excision biopsy with greyish brown outer surface. (C) Cut section shows solid as well as cystic areas.

An excision biopsy was performed which was grossly soft to firm, and nodular measuring 1.5x1.3x1 cm with a greyish-brown outer surface. On cut section it was partly solid and partly cystic (Figure 2). Multiple small cysts each measuring 0.7x0.5x0.5 cm were identified along with brownish-white solid areas.

**Figure 3:**
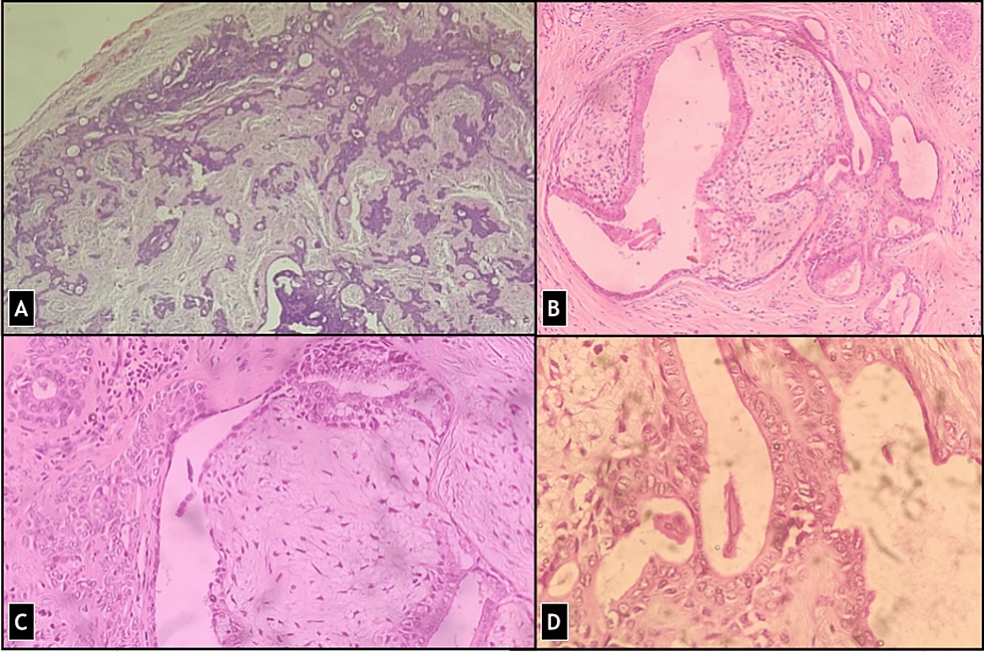
Microscopy shows a well-circumscribed and unencapsulated dermal tumor composed of cells arranged in solid cords, clusters as well as forming ductal structures in chondromyxoid stroma (H&E stain) (A) 40X (B) 100X (C) 200X (D) 400X

**Figure 4:**
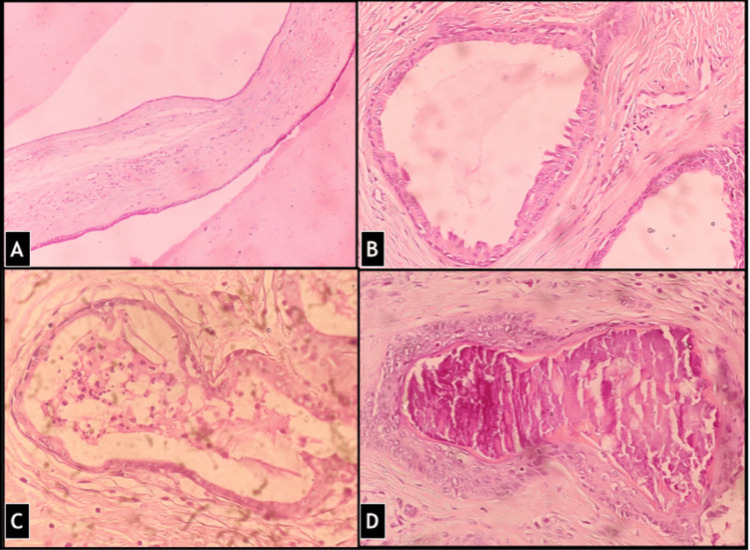
(A) Cystic component was also present forming eccrine hidrocystoma at places (H&E stain, 100 X) (B) Predominantly apocrine glands were seen with few (C) eccrine glands and (D) focal dystrophic calcification (H&E stain, 200X).

**Figure 5:**
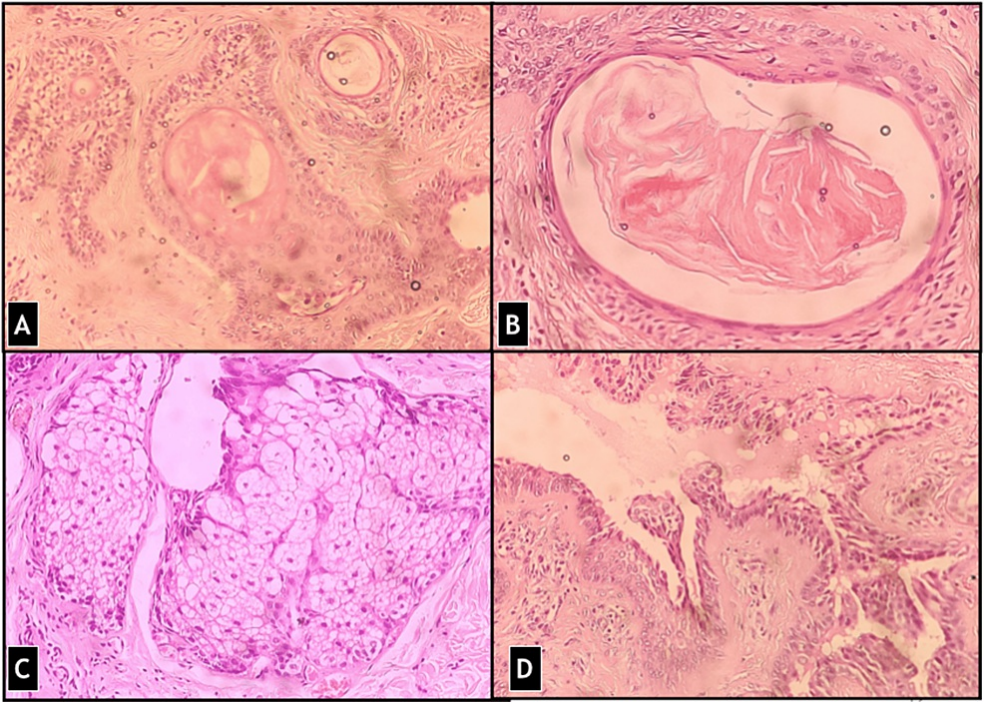
There was presence of focal (A) keratinous cysts (B) glandular secretions in lumen (C) apocrine metaplasia and (D) area of syringocystanedoma papilliferum (H&E stain, 200X).

Sections examined showed a well-circumscribed and unencapsulated benign adnexal tumor composed of cells arranged in solid cords, clusters as well as forming ductal structures showing focal branching and lined by two layers, inner cuboidal and outer cuboidal to flat myoepithelial cells (Figure 3). There were focal nests of polygonal cells with abundant cytoplasm. Some areas showed small round tubules, lined by a single row of epithelial cells (Figure 4). Foci of eccrine hidrocystoma and keratinous or epidermoid cysts were also present (Figure 5). Cholesterol clefts and cystic macrophages were identified in a few cysts. Few islands of squamous epithelium were present. The stroma was chondromyxoid with focal fibromyxoid areas and hyalinization. Focal follicular structures of calcification and adipose metaplasia were seen. A focal component of syringocystadenoma papilliferum was also identified. No cellular atypia, mitosis, areas of haemorrhage or necrosis were seen. Margins were free of tumor cells ensuring complete removal.

Hence it was diagnosed to be a benign adnexal tumor of the skin with features suggestive of chondroid syringoma (mixed tumor of skin) with apocrine as well as focal eccrine features. A component of eccrine hidrocystoma, keratinous cysts and syringocystadenoma papilliferum were also present.

Thereafter patient was evaluated one week postoperatively. There was appropriate local healing without any complication. The patient was explained about the need for regular follow-up visits to monitor recurrence. For the past four months the patient is being observed regularly showing complete healing and no signs of recurrence or complications.

## Discussion

CS or mixed tumor of the skin is an unusual, benign, cutaneous appendageal tumor having both epithelial as well as mesenchymal components [8]. In 1859, Billroth [9] delineated it for the very first time as a category of salivary gland tumors having variable amounts of mucoid and cartilaginous material. In 1961, Hirsch and Helwig [10] established the terminology of “chondroid syringoma” elucidating it as a tumor histopathologically distinguished by the identification of elements of sweat glands lying within a cartilage-like stroma. They also elaborated its five specific histopathologic criteria which included presence of cuboidal or polygonal cell nests; tubuloalveolar structures encircled by two or more than two layers of cuboidal cells; ductal structures comprising of one or two rows of cuboidal cells; occasional keratinous or epidermoid cysts; and a diversified stroma or matrix on hematoxylin and eosin (H&E) stain [10]. Thereafter, Headington [11] explained that CS originates from either eccrine or apocrine glands. Whereas Winkleman and Gottlieb [12], Lever and Shaumburg-Lever [13], and Winkelmann and Muller [14] were affirmative about the eccrine origin of CS in their research studies.

CS is an uncommon lesion having male predominance usually in middle or old age [15,16]. Male to female ratio was 5:1 in the pioneer study by Hirsch and Helwig [10]. It was reported to be 2:1 by Stout and Gorman [17] in their case series of 134 patients of CS. The age range of reported cases is uniformly spread from 23 to 65 years of age with 42 years as the mean age of presentation [3]. It is more commonly seen in middle-aged or elderly men than women [7,18,19]. Nevertheless, it has also rarely been reported in young age [20]. 

It is most frequently localised at the nose, cheek, upper lip, scalp, forehead, and chin in the head and neck region [6,10]. Nasomaxillary groove, eyelids, orbit and rarely foot can also be involved [4,7,21,22]. It can also occur at other sites like the torso, and scrotum [23]. It might localise to any region in the body [3,10]. Although CS is generally benign, a few cases of malignant lesions have also been identified. Such patients are usually younger in age with a male-to-female ratio of 1:2. The extremities and the trunk are the most frequent site of involvement [24]. In the study by Hirsch and Helwig [10], 150 out of 188 cases presented in the head and neck area, in the underlying declining sequence of frequency: nose, cheek, upper lip, scalp, forehead, and chin. A total of 19 cases presented in the extremities, nine in the axilla, eight on the trunk, and two over the genitals [10]. Stout and Gorman [17] studied 134 cases, 66% (89 cases) of which presented in the head and neck [17]. Our case also presented with a tumor in the chin.

The tumor is commonly erythematous, violet or skin coloured, firm, intradermal or subcutaneous, nodule measuring 0.5 to 3 cm in size, usually asymptomatic and growing slowly [3,25-27]. However, larger forms and even multiple tumors of CS have been reported [20,28]. Tumor size of more than 3 cm is related with higher chances of malignancy. Malignant CS classically emerges de novo and not from a preceding benign CS [9]. Patients with these malignant forms commonly consult the clinician after many years of its presence, usually due increase in size or localised injury [25]. 

As a case of CS usually has a firm, round, movable nodule which is going slowly and frequently causes no symptoms, it can be readily interpreted as an epidermal cyst clinically [24]. Nevertheless, some cases of CS with expeditious growth, ulceration, and necrosis have also been reported [9]. The clinical differential diagnoses are sebaceous or dermoid cysts, pilar cyst, clear cell hidradenoma, compound naevus, cystic basal cell carcinoma, neurofibroma and dermatofibroma [1]. The deep-seated form of CS can be clinically diagnosed to be a salivary gland pleomorphic adenoma [29]. Sometimes it can also be confused with a calcifying epithelioma or solitary trichoepithelioma clinically [30]. Hence, due to a wide range of clinical possibilities, a definite diagnosis is usually possible only after excision biopsy [1].

However, FNAC can be done pre-excision, especially in cases with unusual presentation, to diagnose CS. But it has limitations, as CS is a mixed tumor and may show multiple components, and so depending upon the area which is aspirated, only one component can be seen on aspirates. These sampling errors can lead to a misdiagnosis [31]. Like in our case, on FNAC, the sample was aspirated from the area of keratinous cyst and hence it was suggestive of an epidermoid cyst. To reduce these errors, multiple aspirations from different areas of the tumor should be performed.

Histopathologically CS is well-circumscribed with epithelial, myoepithelial, and stromal components [24]. The morphological features of CS are very specific and include presence of tubulo-alveolar and gland-like structures lined by two or more than two cuboidal cell layers, islands of polygonal or cuboidal cells in a chondroid, hyaline, fibroadipose, mucoid or myxoid hypocellular stroma which is periodic acid-Schiff (PAS) and Alcian blue (AB) stain positive [3]. The tubules lined by one or more layers of epithelial cells with eosinophilic cytoplasm form the epithelial element. The flattened myoepithelial cells form the outermost lining of these tubules [24]. The stroma is variable, can show an admixture of different types and occasionally can be osteoid as well [32,33]. Foci of keratinous cysts, squamous differentiation and calcification can be identified [24]. CS being a mixed tumor may show multiple components [31]. Two histopathological types were defined by Headington [11], which include the eccrine variant comprising small round uniform tubules, lined by a single layer of epithelial cells and the apocrine variant showing tubular and cystic branching lumina, which are lined by two layers of epithelial cells.

Histologically, our case fulfilled all the five characteristics of CS as there was presence of nests of polygonal cells; intermixed tubulo-alveolar structures encircled by two and more layers of cuboidal cells; ductal structures lined by one or two layers of cuboidal cells; keratinous cysts; and a chondromyxoid matrix. CS may have few or all of these five characteristics [10]. The tumor in our case was of apocrine as well as focal eccrine type. There were various other components of eccrine hidrocystoma, keratinous cysts and syringocystadenoma papilliferum focally. No cellular atypia was seen and margins were free of tumor.

We studied this case as the clinical diagnosis as well as the FNAC findings were different from the definitive diagnosis on biopsy. The reasons for the same were evaluated and we concluded that this uncommon lesion should always be kept in mind during the clinical as well as cytopathological evaluation. The morphological findings in this case of chondroid syringoma included similar findings to the cases reported earlier in literature. But additional foci of eccrine hidrocystoma as well as syringocystadenoma papilliferum were also identified. In the literature it is mentioned that multiple components to other tumors can be seen in chondroid syringoma, but such a case with various components is rarely reported. And hence it should be remembered that the diagnosis may be missed on FNAC if a single component is aspirated.

The unusual malignant variants increase to a size of more than 3cm and show local invasion. The signs of malignancy on histopathological examination are cytologic atypia, tumor necrosis, infiltrative margins, satellite tumor nodules, and invasion of the deep structures [5,34]. Regional lymph node involvement and bony or visceral metastases can also be present in these cases [1].

Immunohistochemistry (IHC) can be used as an adjunct to support the diagnosis of CS. The tubuloalveolar elements show immunopositivity for cytokeratin (CK), epithelial membrane antigen (EMA), carcinoembryonic antigen (CEA), S-100, neuron-specific enolase (NSE), vimentin, and sometimes glial fibrillary acidic protein (GFAP) [8,35]. The stromal component is focally immunopositive for keratin, desmin, vimentin and S-100 [3]. 

There was a limitation in the number of cases of CS. If we had more cases, we could have studied various clinical presentations and morphological findings in our region. Also, we were unable to do adjunctive immunohistochemistry due to technical considerations.

Treatment of choice for benign CS is surgical excision with a surrounding cuff of normal tissue without affecting functional and esthetic and structures [25]. Electrodessication, dermabrasion and vaporization with argon or carbon dioxide (CO2) laser are other treatment modalities available [3]. The first line of treatment for malignant CS is aggressive surgery. This should be followed by adjuvant radiotherapy with or without chemotherapy [32,33]. A frequent and regular follow-up of the patient is required to evaluate for recurrence locally on the same site or any characteristics of malignancy [36]. In accordance with this, our patient is currently under close follow-up. However, some studies conclude that a long-term follow-up is not required if complete excision of the lesion has been performed and it is histologically proven to be benign. Indication of long-term follow-up is incompletely excised lesion or any features of malignancy [37]. Recurrence is infrequent and occurs due to the tumor being lobulated with an absence of complete capsule leading to its incomplete excision [10,17]. If there is recurrence, a surgical re-excision needs to be done [38].

## Conclusions

As seen in our case, FNAC may mislead in the diagnosis of CS due to its variable components. Excision biopsy is a must for final diagnosis of CS as we can encounter multiple components comprising other lesions as well. Like in our case we encountered a focus of eccrine hidrocystoma, keratinous cysts as well as syringocystadenoma papilliferum. These case findings fit within the existing literature. Hence, in spite of being a rare entity, CS should be considered one of the differential diagnoses of cutaneous nodular swelling in the head and neck region. It is imperative to go for complete excision of the lesion along with a surrounding cuff of normal tissue. Missing the morphological diagnosis can lead to an incompletely excised tumor followed by recurrence. A frequent follow-up of the case is essential even after its complete removal, as it is susceptible to malignant transformation.
